# Action of Chitosan Against *Xanthomonas* Pathogenic Bacteria Isolated from *Euphorbia pulcherrima*

**DOI:** 10.3390/molecules17067028

**Published:** 2012-06-07

**Authors:** Yanli Wang, Liping Li, Bin Li, Guoxing Wu, Qiaomei Tang, Muhammad Ibrahim, Hongye Li, Guanlin Xie, Guochang Sun

**Affiliations:** 1 State Key Laboratory of Rice Biology, Institute of Biotechnology, Zhejiang University, Hangzhou 310058, China; 2 State Key Laboratory Breeding Base for Zhejiang Sustainable Plant Pest and Disease Control, Institute of Plant Protection Microbiology, Zhejiang Academy of Agricultural Sciences, Hangzhou 310021, China; 3 Institute of Bioinformatics, Zhejiang University, Hangzhou 310058, China; 4 College of Plant Protection, Yunnan Agricultural University, Kunming 650201, China

**Keywords:** antibacterial mechanism, cell membrane, chitosan, biofilm, *Xanthomonas*

## Abstract

The antibacterial activity and mechanism of two kinds of chitosan were investigated against twelve *Xanthomonas* strains recovered from *Euphorbia pulcherrima*. Results indicated that both chitosans markedly inhibited bacterial growth based on OD loss. Furthermore, the release of DNA and RNA from three selected strains was increased by both chitosans. However, the release of intracellular proteins was inhibited by both chitosans at different concentration and incubation times, except chitosan A at 0.1 mg/mL for 0.5 h incubation and 0.2 mg/mL for 2.0 h incubation increased the release of proteins, indicating the complexity of the interaction and cell membranes, which was affected by incubation time, bacterial species, chitosan type and concentration. Transmission electron microscopic observations revealed that chitosan caused changes in protoplast concentration and surface morphology. In some cells, the membranes and walls were badly distorted and disrupted, while other cells were enveloped by a thick and compact ribbon-like layer. The contrary influence on cell morphology may explain the differential effect in the release of material. In addition, scanning electron microscope and biofilm formation test revealed that both chitosans removed biofilm biomass. Overall, this study showed that membrane and biofilm play an important role in the antibacterial mechanism of chitosan.

## 1. Introduction

Chitosan is a natural polysaccharide derived by *N*-deacetylation of chitin, a major component of the shells of crustacea such as crab, shrimp, and crawfish [1,2,3,4]. In recent years, chitosan and its derivatives have been getting more and more attention in sustainable agriculture and food safety [5,6,7,8]. Much of the interest in the new applications of chitosan and its derivatives for agriculture has focused on its potential effects in its reported antimicrobial properties such as antivirus, antifungal and antibacterial activities [9]. Indeed, chitosan has several advantages over other types of antimicrobial agents for its higher antimicrobial activity, a broader spectrum of activity, a higher killing rate, and lower toxicity toward mammalian cells [10,11,12,13].

Several studies on antibacterial activities of chitosan against plant associated bacteria have been recently carried out [11,12,13]. In particular, in our previous study chitosan showed strong *in vitro* antibacterial activity against *Xanthomonas* pathogenic bacteria isolated from *Euphorbia pulcherrima* based on the conventional colony count method [2]. However, little information is available about antibacterial mode of action of chitosan against plant pathogenic bacteria. Researchers have applied multiple techniques to investigate chitosan’s antibacterial mode of action [14,15,16,17]. It is generally assumed that the cationic nature of chitosan, conveyed by the positively charged NH_3_^+^ groups of glucosamine, might be a fundamental factor contributing to its interaction with the negatively charged microbial cell surface, ultimately resulting in impairment of vital bacterial activities [18,19,20].

This charge interaction can alter bacterial surface morphology, which either increased membrane permeability in some bacteria, or decreased membrane permeability in other bacteria [17,20,21]. Furthermore, visual confirmation of an effective membrane destruction of chitosan and its derivative has been reported [15,16,19]. In addition, Carlson *et al.* [22] and Martinez *et al.* [23] found that chitosan-coated surfaces have anti-biofilm properties *in vitro* against certain bacteria and fungi. In contrast, several studies indicated that chitosan’s effect might not be as dramatic as lysing bacterial cells [10,17,24], indicating the complexity of antibacterial mechanism of chitosan.

The objective of this research was to examine the role of cell membranes and biofilms in the antibacterial activities of two kinds of chitosan solution against *Xanthomonas* pathogenic bacteria isolated from *E. pulcherrima*.

## 2. Results and Discussion

### 2.1. Molecular Weight and Deacetylation Degree

Although both chitosans were extracted from crab shells, a difference in molecular weight (Mw) and deacetylation degree (DD) values between the two kinds of acid-soluble chitosan was observed. Results from this study indicated that the Mw of chitosan A was about 1,129 kDa, while the Mw of chitosan B was about 607 kDa. The DD of chitosan A was 85.3%, while the DD of chitosan B was 72.0%. However, the instructions indicated that the DDs of chitosan A and B were no less than 85% and 75%, respectively.

### 2.2. Antibacterial Activity of Chitosan

Chitosan type and bacterial species significantly affected the surviving cell numbers (*P* < 0.001; Table 1). In addition, the two-factor interaction between chitosan type and bacterial species was significant (*P* < 0.001; Table 1). This results revealed that the growth of twelve *Xanthomonas* strains were inhibited by chitosan regardless of the kinds of chitosan and the species of bacteria, which implies that the two kinds of chitosan were good bactericide for the control of bacterial disease of *E. pulcherrima*.

**Table 1:** Effect of chitosan solution on the growth of *Xanthomonas* sp. pathogenic to *Euphorbia pulcherrima* from different concentration from geographical sources.

Strain number	Species identity	Strain source	OD_600_ reduction relative to the control (%)	
			Chitosan A	Chitosan B
LMG849	*X. axonopodis* pv. *poinsettiicola*	India	24.20 ± 2.32 cd	20.94 ± 2.29 c
LMG5401	*X. axonopodis* pv. *poinsettiicola*	India	49.95 ± 4.48 f	52.52 ± 3.21 e
LMG5402	*X. arboricola* pv. *poinsettiicola*	New Zealand	61.88 ± 6.07 g	14.35 ± 2.04 ab
LMG5403	*X. arboricola* pv. *poinsettiicola*	New Zealand	53.50 ± 4.74 fg	15.40 ± 1.61 abc
LMG8675	*X. arboricola* pv. *poinsettiicola*	New Zealand	14.40 ± 1.25 ab	16.35 ± 1.25 abc
LMG8676	*X. arboricola* pv. *poinsettiicola*	New Zealand	17.11 ± 1.77 abc	16.07 ± 1.94 abc
R22578	*X. axonopodis* pv. *poinsettiicola*	Hangzhou, China	17.91 ± 1.61 abc	19.75 ± 1.22 bc
R22579	*X. axonopodis* pv. *poinsettiicola*	Hangzhou, China	39.47 ± 4.16 e	13.12 ± 1.28 a
R22580	*X. axonopodis* pv. *poinsettiicola*	Hangzhou, China	30.52 ± 3.27 de	39.51 ± 3.16 d
HN-1	*X. axonopodis* pv. *poinsettiicola*	Hainan, China	11.17 ± 1.27 a	34.53 ± 2.29 d
HN-18	*X. axonopodis* pv. *poinsettiicola*	Hainan, China	23.32 ± 2.43 bcd	34.05 ± 2.21 d
HN-20	*X. axonopodis* pv. *poinsettiicola*	Hainan, China	9.26 ± 1.10 a	16.45 ± 1.45 abc
LSD_0.05_			9.33	6.13
ANOVA *P*-values				
Chitosan			<0.001	
Bacteria			<0.001	
Chitosan × Bacteria			<0.001	

Initial concentration of bacteria is approximately 10^7^ cfu/mL. Data from the repeated experiment were pooled and presented as means ± standard error. Means in a column followed by the same letter are not significantly different according to LSD test (*P* = 0.05).

Results from this study indicated that the addition of chitosan A at 0.10 mg/mL to twelve bacterial strains caused the reduction in the OD_600nm_ after 24 h of incubation. The reduction percentage of chitosan A in the OD_600nm_ ranged from 9.26% to 61.88% as compared to the control while the reduction percentage in strain LMG 5402 and LMG 5403 was more than 50.00% (Table 1). Similarly, the addition of chitosan B at 0.10 mg/mL to twelve bacterial strains caused a reduction in the OD_600nm_ after 24 h of incubation. The reduction percentage in the OD_600nm_ ranged from 13.12% to 52.52% as compared to the control while the reduction percentage in strain LMG 5401 was more than 50.00% (Table 1). This result was consistent with our previous result, which found chitosan A at 0.20 mg/mL had strong antibacterial activity against nine strains of *X. arboricola* pv. *poinsettiicola *and *X. axonopodis* pv. *poinsettiicola* from different geographic sources based on the colony count method [2]. However, in this study the twelve *Xanthomonas* strains in general showed difference in the sensitivity to two kinds of chitosan. In particular, the reduction percentage of chitosan B in the OD_600nm_ of strain LMG 5402 and LMG 5403 was 14.35% and 15.40%, respectively, while the reduction percentage of chitosan A in the OD_600nm_ of strain LMG 5402 and LMG 5403 was 61.88% and 53.50%, respectively, as compared to the corresponding control. In contrast, strain LMG 5401 showed high susceptibility to both chitosan A and B, which caused the reduction in OD_600nm_ by 49.95% and 52.52%, respectively, as compared to the control. The difference in the sensitivity of bacteria to chitosan may be attributed to the complexity of interaction between the two kinds of chitosan and these *Xanthomonas* strains.

### 2.3. Antibacterial Mechanism of Chitosan

#### 2.3.1. Integrity of Cell Membrane

Chitosan type and incubation time significantly affected the OD_260 _values of strain R22579 (*P *< 0.001; Table 2). However, there was no interaction between chitosan type and incubation time (*P *= 0.56; Table 2). 

**Table 2:** The ratio release (*vs.* control) of cell materials absorbing at 260 nm over time from *Xanthomonas* strain R22579 treated with chitosan at different concentration.

Chitosan (mg/mL)		The ratio release ( *vs.* control) at OD_260_ (%)			
		0.5 h	1.0 h	1.5 h	2.0 h
A					
	0.1	104.05 ± 0.03 b	103.74 ± 0.01 a	103.07 ± 0.04 b	103.23 ± 0.04 b
	0.2	104.24 ± 0.04 c	104.32 ± 0.04 c	103.65 ± 0.02 c	104.20 ± 0.04 d
	0.3	103.08 ± 0.03 a	104.09 ± 0.05 b	102.66 ± 0.08 a	103.65 ± 0.07 c
	0.4	103.02 ± 0.04 a	104.15 ± 0.08 b	102.76 ± 0.02 a	102.97 ± 0.04 a
	0.5	104.92 ± 0.02 c	105.82 ± 0.03 d	105.56 ± 0.05 d	106.33 ± 0.02 e
B					
	0.1	103.77 ± 0.01 e	104.44 ± 0.05 c	103.06 ± 0.03 c	103.67 ± 0.03 d
	0.2	103.34 ± 0.03 d	104.34 ± 0.06 c	103.18 ± 0.07 c	103.54 ± 0.07 d
	0.3	101.99 ± 0.02 b	103.53 ± 0.06 b	102.64 ± 0.04 b	103.01 ± 0.05 c
	0.4	102.28 ± 0.01 c	103.13 ± 0.07 a	102.29 ± 0.10 a	102.48 ± 0.06 a
	0.5	101.62 ± 0.01 a	103.21 ± 0.02 a	102.45 ± 0.04 ab	102.82 ± 0.06 b
LSD_0.05_		0.08	0.15	0.16	0.15
ANOVA *P*-values					
Chitosan			<0.001		
Incubation time			<0.001		
Chitosan × Incubation time			0.56		

Initial concentration of bacteria is approximately 10^7^ cfu/mL. Data from the repeated experiment were pooled and presented as means ± standard error. Treatments within each group of chitosan with different letters are significantly different according to LSD test (*P* = 0.05).

In general, in presence of chitosan, the release of DNA and RNA from strain R22579 were increased by 1.62% to 6.33% compared to the control, regardless of chitosan type, chitosan concentration and incubation time (Table 2). However, chitosan A had a greater increase in the OD_260_ values compared to that of chitosan B. In addition, the highest increase in the OD_260_ values by chitosan was obtained after 1.0 h of incubation.

The OD_280_ values of strain R22579 was significantly affected by chitosan type (*P* < 0.001; Table 3), but unaffected by incubation time (*P *= 0.65; Table 3). In addition, there was no interaction between chitosan type and incubation time (*P *= 0.49; Table 3). Chitosan A at 0.1 mg/mL increased the releases of intracellular proteins after 0.5, 1.0 and 1.5 h of incubation while chitosan A at 0.2 mg/mL increased the releases of intracellular proteins after 1.0, 1.5 and 2.0 h of incubation. However, the releases of intracellular proteins was inhibited by 0.17% to 7.76% in all other treatments compared to the control regardless of chitosan type, chitosan concentration and incubation time (Table 3). In general, chitosan B showed greater inhibition in the OD_280_ values compared to that of chitosan A. In addition, the highest increase by chitosan was obtained after 0.5 h of incubation.

**Table 3:** The ratio release (*vs.* control) at OD_280_ (%) from *Xanthomonas* strain R22579 treated with chitosan at different concentrations.

Chitosan (mg/mL)		The ratio release at OD_280_				
		0.5 h		1.0 h	1.5 h	2.0 h
A						
	0.1	99.64 ± 0.03 c		100.49 ± 0.06 d	100.25 ± 0.07 d	100.30 ± 0.01 d
	0.2	100.58 ± 0.01 d		102.01 ± 0.02 e	100.88 ± 0.08 e	99.83 ± 0.03 c
	0.3	98.87 ± 0.02 b		99.63 ± 0.16 c	99.11 ± 0.14 c	98.77 ± 0.02 b
	0.4	98.32 ± 0.01 a		98.14 ± 0.04 a	97.97 ± 0.07 a	97.37 ± 0.06 a
	0.5	98.32 ± 0.06 a		98.48 ± 0.08 b	98.72 ± 0.07 b	98.84 ± 0.15 b
B						
	0.1	92.24 ± 0.10 a		92.59 ± 0.04 a	93.33 ± 0.08 a	94.23 ± 0.06 a
	0.2	97.61 ± 0.04 d		98.18 ± 0.01 c	98.61 ± 0.09 d	98.11 ± 0.02 c
	0.3	97.38 ± 0.03 c		97.26 ± 0.06 b	98.27 ± 0.04 c	98.79 ± 0.01 e
	0.4	98.15 ± 0.09 e		98.35 ± 0.03 d	98.25 ± 0.04 c	98.30 ± 0.05 d
	0.5	96.70 ± 0.01 b		97.26 ± 0.02 b	97.27 ± 0.04 b	96.76 ± 0.08 b
LSD_0.05_		0.15		0.13	0.22	0.14
ANOVA *P*-values						
Chitosan			<0.001			
Incubation time			0.65			
Chitosan × Incubation time			0.49			

Initial concentration of bacteria is approximately 10^7^ cfu/mL. Data from the repeated experiment were pooled and presented as means ± standard error. Treatments within each group of chitosan with different letters are significantly different according to LSD test (*P* = 0.05).

Resulsts from this study indicated that chitosan at higher than 0.3 mg/mL increased the release of DNA and RNA, but inhibited the release of intracellular proteins from strain R22579 compared to the control regardless of chitosan type, chitosan concentration and incubation time, which may be attributed to the difference in structure and molecular size of between DNA, RNA and intracellular proteins. In addition, the similar result on the release of DNA, RNA and intracellular proteins were obtained from strains LMG 849 and HN-18 (data not shown).

It is believable that positive charge chitosan can interact with the negative charge bacterial membrane, which may cause the leaching out of low molecular weight materials, nucleic acid, proteins and so on [24,27,30]. However, several recent studies have also shown that this charge interaction can alter bacterial surface morphology, which either increases membrane permeability, causing leakage of intracellular substances, or decreases membrane permeability, preventing nutrient transport, indicating the complexity of the interaction between chitosan and bacterial membrane [15,16,17].

In general, this study showed that the effect of chitosan A on the release of DNA, RNA and intracellular proteins is similar with that of chitosan B. However, the release of intracellular proteins was inhibited by chitosan A at 0.1 for 0.5 h of incubation and at 0.2 mg/mL for 2.0 h of incubation while was inhibited by chitosan B regardless of chitosan concentration and incubation time, which may be attributed to the difference in Mw and DD values between two kinds of acid-soluble chitosan. In addition, the result of antibacterial activity indicated that chitosan A at 0.1 mg/mL caused more reduction in the cell numbers compared to that of chitosan B, which may partially explain the result that chitosan A and B at 0.1 and 0.2 mg/mL had differential effects in the release of intracellular proteins.

#### 2.3.2. Bioﬁlm Formation

Biofilm formation was significantly affected by bacterial species (*P*<0.001; Table 4), but unaffected by chitosan type. In addition, the two- factor interaction between chitosan type and bacterial species was significant (*P* < 0.001; Table 4). In agreement with the result of antibacterial activity of chitosan, the two kinds of chitosan at 0.10 mg/mL reduced the formation of bacterial biofilm after 24 h of incubation relative to the corresponding control regardless of the tested strain (Table 4). The reduction percentage of chitosan A in biofilm formation ranged from 7.31% to 32.96% while the reduction percentage of chitosan B ranged from 9.47% to 42.52% after 24 h of incubation (Table 4). The result revealed that the destruction of biofilm formation may play an important role in antibacterial activity of the two kinds of chitosan against *Xanthomonas* pathogenic bacteria.

Biofilm formation in plant pathogenic bacteria that are strongly related to disease is a very active field of research [31]. By attachment to submerged surfaces and production of microbial products such as polysaccharides, bacteria are able to form biofilm with complex, three-dimensional structures [31,32]. The top or base of the biofilm has the highest cell density, and water channels exist for the transport of nutrients and waste [31,33]. Interestingly, result from this study indicated that chitosan was a good inhibitor of bacterial biofilm formation.

In general, the biofilm formation of these *Xanthomonas* strains was differentially reduced by the two kinds of chitosan, which may be attributed to the difference in physiological state of bacteria and the kind of chitosan. In addition, a close correlation was not observed between the reduction of cell membrane and the reduction of biofilm formation, which revealed that other factors other than biofilm formation may be also involved in the antibacterial mechanism of chitosan. Indeed, the dead cells may help biofilm reformation during the early formation of biofilm, which may partially explain the result that the reduction of biofilm formation is lower than that of the cell numbers in most bacterial strains.

**Table 4:** Effect of chitosan on biofilm formation of *Xanthomonas* strains after 24 h of incubation.

Strain number	OD_570_ reduction relative to the control (%)		
	Chitosan A		Chitosan B
LMG849	18.05 ± 2.02 de		28.56 ± 3.43 ef
LMG5401	32.96 ± 3.52 g		21.25 ± 2.37 cd
LMG5402	27.53 ± 2.19 fg		22.15 ± 3.46 de
LMG5403	28.70 ± 2.18 g		14.91 ± 1.77 abc
LMG8675	7.31 ± 1.04 a		16.79 ± 1.76 bcd
LMG8676	9.36 ± 1.26 ab		11.68 ± 1.13 ab
R22578	14.13 ± 1.60 bcd		10.37 ± 1.72 ab
R22579	9.72 ± 0.64 ab		11.73 ± 1.07 ab
R22580	10.90 ± 1.04 abc		9.47 ± 0.84 a
HN-1	15.47 ± 1.75 cd		12.82 ± 1.63 ab
HN-18	28.09 ± 2.17 fg		42.52 ± 3.57 g
HN-20	23.07 ± 1.96 ef		35.09 ± 2.96 f
LSD_0.05_	5.51		6.84
ANOVA *P*-values			
Chitosan		0.25	
Bacteria		<0.001	
Chitosan × Bacteria		<0.001	

The concentration of chitosan is 0.10 mg/mL. Initial concentration of bacteria is 10^7^ cfu/mL. Data from the repeated experiment were pooled and presented as means ± standard error. Means in a column followed by the same letter are not significantly different according to LSD test (*P* = 0.05).

#### 2.3.3. SEM

**Figure 1:**
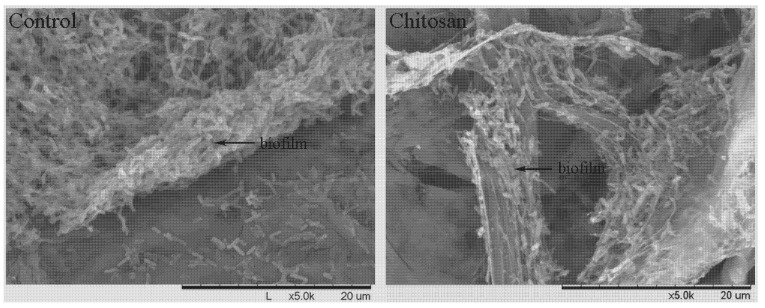
Scanning electron microphotographs of *Xanthomonas axonopodis* pv. *poinsettiicola* strain R22579 treated with sterile distilled water (control) and with 0.10 mg/mL chitosan A. Bar: μm.

In scanning electron micrographs, the control show thick, intact and compact structured biofilm (Figure 1). The bacterial cells gathered and formed adhesion layers. The secretions which were sticky and make bacterial cells adhere to one another were homogeneous and nonopaque. By contrast, the samples treated with chitosan appeared thin, fragmentized, non-uniform and with albuminous degeneration. The chitosan interacted with extracellular secretions to form light-proof flocs. The flocs seem compact, sticky and bounded the bacterial cells. It can be observed that the chitosan destroyed the structure of the biofilm, which inferred that the degeneration of organic matter caused by chitosan would damage the physiological function of biofilm.

Result from this study indicated that the two kinds of chitosan were able to destroy bacterial bioﬁlm formation, which is in agreement with the data of biofilm biomass of these *Xanthomonas* strains. It is well known that bacterial bioﬁlm is very important for helping bacteria to resist environmental stress [31,32]. Without the protection of biofilm, the bacterial cells are more feasible to be killed by bactericide at lower concentration. Therefore, antibacterial activity of the two kinds of chitosan against these *Xanthomonas* strains may at least in part be attributed to the damage of biofilm formation.

#### 2.3.4. TEM

**Figure 2:**
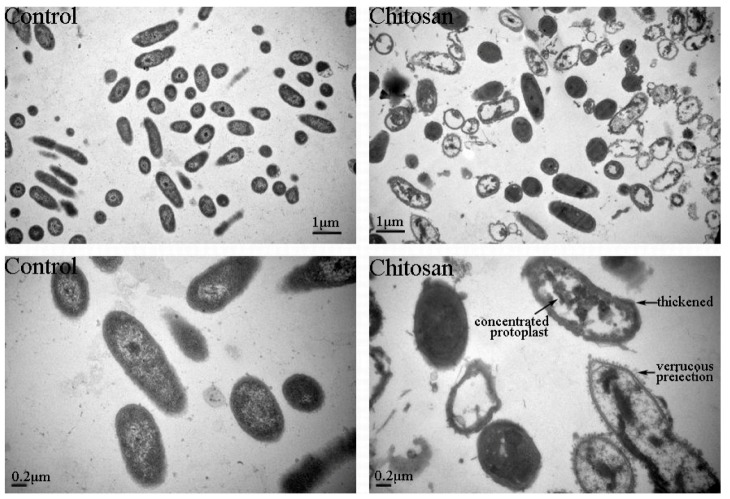
Transmission electron microphotographs of *Xanthomonas axonopodis* pv. *poinsettiicola* strain R22579 treated with sterile distilled water (control) and with 0.10 mg/mL chitosan A. Bar: μm.

In transmission electron micrographs, the control showed intact and apparent cell membranes with uniformly distributed cytochylema and electron density inside the cell while concentrated protoplasts and the altered surface morphology were observed in almost all cells of strain R22579 treated with chitosan A (Figure 2). However, binding of chitosan resulted in a differential change in bacterial surface morphology. In some cells, the membranes and walls were badly distorted and even disrupted, which may increase membrane permeability and cause leakage of intracellular substances, while other cells were enveloped by a thick and compact ribbon-like layers, which may decrease membrane permeability and impede the interchange of materials between the inner and outer of the cell.

In general, this data is consistent with the result of integrity of cell membranes test, which found that chitosan had differential effect in the release of DNA, RNA and intracellular proteins from bacterial strains. However, in contrast with the result of this study, TEM images revealed that the intracellular materials were not condensed in cells of *Escherichia coli* although chitosan caused the disruption of the outer membrane structure with membrane sloughing and breaching, even disappearing, which resulted in almost all the intracellular materials being directly suspended in chitosan solution [27].

Binding of chitosan to bacterial cell surface either increased membrane permeability, causing leakage of intracellular substances, or decreased membrane permeability, preventing nutrient transport [17,18,21,22]. However, results from this study revealed that the cells in the same kind of bacteria had different reactions to chitosan in terms of TEM observation, indicating the complexity of interaction between the positively charged chitosan and the negatively charged bacterial membrane. The difference may be attributed to the difference in the physiological state of bacterial cells. In addition, bacterial cells may have different responses to the environmental pressure, which may in part result in the diversity change in surface morphology of bacterial cells.

## 3. Experimental

### 3.1. Preparation of Chitosan Stock

Chitosan with degrees of *N*-deacetylation no less than 85% (practical grade, from crab shells) and 75% (from crab shells) were applied and obtained from Sigma-Aldrich (St. Louis, MO, USA), and named as chitosan A and chitosan B, respectively. Stock solutions of chitosan A or B (5 mg/mL) were prepared in 1% acetic acid with the pH being adjusted to 6.0 with NaOH [2]. After stirring (160 rpm) for 24 h at room temperature, the stock solution was autoclaved at 121 °C for 20 min. Sterile deionized water of pH 6.0 was used as a control.

### 3.2. Molecular Weight and Deacetylation Degree

The Mw of chitosan were measured by Center of Analysis & Measurement of Zhejiang University using Gel Permeation Chromatography (GPC). The DD of chitosan were determined using the method described by Muzzarelli and Rocchetti [25].

### 3.3. Bacterial Strains

To obtain the antibacterial activity of chitosan, twelve strains of *Xanthomonas* that cause leaf spot disease of *E. pulcherrima* were collected from India, New Zealand, and China (Table 1). All bacterial strains involved in this study were deposited in both the culture collection of the Laboratorium voor Microbiologie, Universiteit Gent, Gent, Belgium (LMG) and the Institute of Biotechnology, Zhejiang University, China. The bacterial strains were cultured on nutrient agar medium [2] at 28 °C. After 48 h of incubation, each bacterial suspension was prepared in Luria-Bertani broth (LB).

### 3.4. Inhibition Assessment

The inhibition activity of both chitosans against *Xanthomonas* pathogenic bacteria was evaluated by measuring optical density (OD) 600, as described by Li *et al. *[26]. *Xanthomonas* strains were cultivated in nutrient agar and incubated at 28 °C. A representative colony was picked off and placed in plus-LB (peptone 10 g, yeast extract powder 5 g, NaCl 5 g, distilled water 1,000 mL, glucose 1 g, pH 7.2) and incubated overnight on the rotary shaker (Hualida Company, Taicang, China) at 160 rpm, 28 °C. Chitosan stock was added to the cultured bacterial suspension to give a final concentration of either chitosan A or B at 0.1 mg/mL and then the mixture was incubated at 28 °C on a rotary shaker at 160 rpm for six hours while the same volume of sterile distilled water was added to the controls. After that, 200 μL of the mixture was filled to each well of 96-well plate (commercially available presterilized, polystyrene, ﬂat-bottom). Each treatment consists of twelve wells. The plates were incubated at 28 °C without shaking. After 24 h, the plates were read by a microplate reader (Thermo Fisher Scientific Inc., Waltham, MA, USA) at 600 nm. 

### 3.5. Integrity of Cell Membrane

Cell membrane integrity of *Xanthomonas* strains R22579, LMG 849 and HN-1 were examined by determination of the release of material absorption value at 260 nm [14] and 280 nm [27]. The cultured bacteria were harvested by centrifuging at 5,000 rpm for 5 min and washed twice then resuspended in sterile phosphate buffer saline. The ﬁnal cell suspension was adjusted to an absorbance at 630 nm (OD_630_) of 0.6 and OD_420_ of 0.8 to measure the OD_260_ and OD_280_, respectively. The chitosan solutions were added to bacterial suspension to give a final chitosan concentration of 0.1, 0.2, 0.3, 0.4 and 0.5 mg/mL, and the releases over time (0.5, 1.0, 1.5 and 2.0 h) of materials absorbing at 260 and 280 nm were recorded with a lambda 35 uv/vis spectrophotometer (PerkinElmer, Norwalk, CT, USA).

### 3.6. Bioﬁlm Formation

The formation of *Xanthomonas* bioﬁlm was studied in polystyrene 96-well microtitre plates based on the method described previously [28,29] with some modifications. Chitosan stock was added to the overnight bacterial suspension to give a final concentration of chitosan of 0.1 mg/mL while the same volume of sterile distilled water was added to the controls. The mixture was incubated for six hours following that 200 μL of the suspension was added to individual wells of polystyrene 96-well plates and incubated at 28 °C without shaking. Each treatment consists of twelve wells. Bioﬁlm was cultured for 24 h and then the wells were washed three times with sterile distilled water to remove non-adhered bacteria. The remaining attached bacteria were air-dried and stained with 1% (w/v) crystal violet solution of 250 μL per well at room temperature for 20 min. The wells were washed to remove non-adsorbed crystal violet solution and immediately de-stained with 200 μL of 33% acetic acid. The de-staining solution was measured with microplate reader (Thermo Fisher Scientific Inc., Waltham, MA, USA) at 570 nm.

### 3.7. Scanning Electron Microscope (SEM)

Bioﬁlm of *Xanthomonas* strain R22579 was grown on weighing papers (5 mm × 5 mm) in centrifuge tubes with plus-LB at 28 °C for 3 days. Chitosan stock was added to the overnight bacterial suspension to give a final chitosan concentration of 0.1 mg/mL while the same volume of sterile distilled water was added to the controls. The mixed solutions were incubated for 6 h. Weighing papers with bioﬁlm were washed three times with phosphate buffer and transferred to another centrifuge tubes containing 2.5% glutaraldehyde and incubated overnight at 4 °C. Then, the samples were washed with phosphate buffer three times, 10 min for one time and serially dehydrated in a graded series of alcohol (50, 70, 80, 90, 95 and 100%, v/v). Finally, the samples were ﬁxed in a criticalpoint drier, coated with gold-palladium, and viewed with a JEOL (Tokyo, Japan) JSM-6400 scanning electron microscope.

### 3.8. Transmission Electron Microscope (TEM)

*Xanthomonas* strain R22579 was prepared for TEM as previously described [10,27]. Chitosan stock was added to the *Xanthomonas* suspension cultivated for three days to give a ﬁnal concentration of chitosan of 0.1 mg/mL while the same volume of sterile distilled water was added to the controls. After 6 h treatment, the *Xanthomonas* suspension was centrifuged and the cells were washed and transferred to another centrifuge tubes containing 2.5% glutaraldehyde and incubated overnight at 4 °C. Then, the samples were serially dehydrated in a graded series of ethanol solutions (70, 80, 90 and 100%, v/v), then post-ﬁxed with 1% (w/v) OsO_4_ for 1 h at room temperature, and washed three times. After that, the samples were embedded in agar. Thin sections of the specimens were cut with a diamond knife on an Ultracut Ultramicrotome (Super Nova; Reichert-Jung Optische Werke, Wien, Austria) and the sections were double-stained with saturated uranyl acetate and lead citrate. The grids were examined with a JEM-1230 transmission electron microscope (Hitachi, Tokyo, Japan) at an operating voltage of 75 kV.

### 3.9. Statistical Analysis

The software STATGRAPHICS Plus, version 5.1 (Copyright Manugistics Inc., Rockville, MD, USA) was used to perform the statistical analysis. Levels of significance of the main factors and their interactions were calculated by two-way analysis of variance after testing for normality and variance homogeneity.

## 4. Conclusions

In this study, two kinds of chitosan solutions at 0.1 mg/mL markedly inhibited the growth of the twelve *Xanthomonas* strains from different geographical origins by measuring the OD value at 600 nm. The reduction in the OD of cell suspension depends on the type of chitosan and the species of bacteria. Results from this study indicated that the two kinds of chitosans removed biofilm biomass of *Xanthomonas* strains based on SEM observation and biofilm formation test, indicating the importance of biofilm in antibacterial activity of chitosan. In general, chitosan increased the release of DNA and RNA, but inhibited the release of intracellular proteins from bacteria based on an integrity of cell membranes test. Further, TEM observations found that binding of chitosan leads to protoplast concentration. However, membranes and walls became distorted and ruptured in some cells to release intracellular materials but became thickened in another cells to block-up the regular interchange of materials through the membrane. This indicated the complexity of the interactions between chitosan and bacterial membranes. Overall, these results revealed that the antibacterial mode of action of chitosan may be involved in a number of events that may ultimately lead to a killing effect.
